# Instructive interaction between myelodysplastic hematopoiesis and the bone marrow microenvironment at the single-cell level^∗^

**DOI:** 10.1016/j.bneo.2024.100021

**Published:** 2024-05-21

**Authors:** Johann-Christoph Jann, Nanni Schmitt, Alexander Streuer, Qingyu Xu, Vladimir Riabov, Eva Altrock, Nadine Weimer, Verena Nowak, Julia Obländer, Iris Palme, Melda Göl, Marie Demmerle, Felicitas Rapp, Fabian Siegel, Laurenz Steiner, Mahmoud Ghazal, Angelika Duda, Verena Haselmann, Ali Darwich, Ahmed Jawhar, Mohamad Jawhar, Georgia Metzgeroth, Wolf-Karsten Hofmann, Daniel Nowak

**Affiliations:** 1 Department of Hematology and Oncology, Medical Faculty Mannheim, Heidelberg University, Mannheim, Germany; 2 Department of Biomedical Informatics, Medical Faculty Mannheim, Heidelberg University, Mannheim, Germany; 3 Department for Clinical Chemistry, Medical Faculty Mannheim, Heidelberg University, Mannheim, Germany; 4 Department of Orthopedic Surgery, Medical Faculty Mannheim, Heidelberg University, Mannheim, Germany

## Abstract

Myelodysplastic neoplasms (MDS) are hypothesized to remodel their bone marrow (BM) microenvironment to reinforce conditions for their propagation. In this study, we investigated interactions between MDS cells and the BM niche at single-cell level. In a patient-derived xenograft (PDX) model, we analyzed 13 000 cells from different murine niche cell populations after long-term (>24 weeks) exposure to MDS vs healthy human grafts. Subsequently, we analyzed over 24 000 primary human BM cells enriched for the nonhematopoietic compartment by using whole bone fragments from n = 8 patients with MDS and n = 7 healthy, age-matched donors. In PDX who received MDS transplantation, mesenchymal cell (MSC) subpopulations were forced to overexpress hematopoietic factors such as Cxcl12 and Il7 upon contact with hematopoietic MDS cells as compared with healthy grafts. Single-cell analyses of primary in situ BM cells from patients with MDS showed highly heterogeneous MSC subpopulations on a patient-individual level. We identified inflammatory gene expression profiles as well as overexpression of C-X-C Motif Chemokine Ligand 12, KIT ligand, and Interleukin 7 in MDS MSCs and endothelial cells. In conclusion, we demonstrate reprogramming of the BM microenvironment by MDS cells, pointing to altered MSC subpopulations with increased growth factor expression profiles in a subgroup of patients with MDS.

## Key Points

• BM stroma cells of primary and xenografted MDS overexpress hematopoietic supporting factors.
• The primary mesenchymal niche in MDS exhibits significant patient-level heterogeneity.

## Visual Abstract

**Figure:**
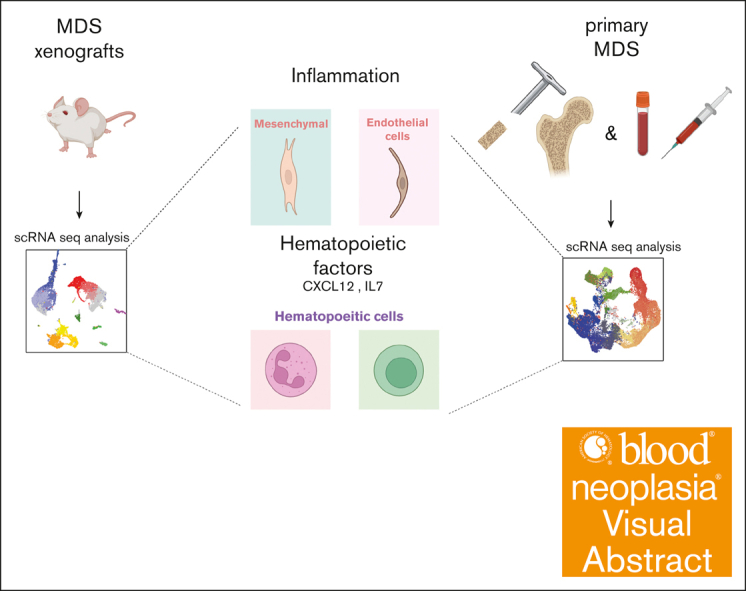

## Introduction

Myelodysplastic neoplasms (MDS) are heterogeneous clonal malignancies of hematopoiesis characterized by impaired hematopoietic differentiation resulting in peripheral cytopenia. In MDS, a multitude of disease-associated molecular aberrations have been discovered within the hematopoietic compartment. However, it is not yet fully understood how these interact and depend on cell extrinsic factors, such as inflammation and aging, to ultimately translate into the pathogenesis of MDS.^1^ Increasing evidence has also suggested that MDS may not only be a disease of hematopoiesis but also of the surrounding bone marrow (BM) microenvironment,^2^ also termed the BM niche or stroma. An active role of the BM niche in MDS but also in other myeloid neoplasms, such as myeloproliferative neoplasms or acute myeloid leukemia (AML), has been demonstrated in several experimental approaches.^3^, ^4^, ^5^, ^6^ Studies in mice have recently led to the definition of nonhematopoietic cell populations in the BM and have shown how these specifically influence hematopoietic function.^7^, ^8^, ^9^, ^10^, ^11^, ^12^ However, the primary human BM niche, in particular that of patients with MDS, and its interaction with hematopoietic progenitor cells are much less studied. Furthermore, in primary settings of MDS, it is not yet clear whether phenotypic changes in the BM stroma are the cause or consequence of alterations in the hematopoietic compartment.

We therefore aim to discover and annotate interactions in the primary human BM niche derived from clinical samples from patients with MDS. We enriched for nonhematopoietic cells from intact bone fragments and compared them with samples from donors without evidence of myeloid neoplasia or clonal hematopoiesis. We investigated the cellular composition of the murine and human BM stroma by using single-cell RNA sequencing and cellular indexing of the transcriptome and epitopes by sequencing (CITE-seq) to specifically profile both unselected and enriched nonhematopoietic BM cells to answer the question of the stromal response to MDS hematopoiesis using an unbiased approach.

## Methods

### Human studies

All human data presented here were generated after patients’ written informed consent to procedures in accordance with the Declaration of Helsinki and approved by the medical ethics committee II of the Medical Faculty Mannheim, Heidelberg University, Heidelberg, Germany. All patient samples were obtained from residual specimens from diagnostic BM biopsies at the Department of Hematology and Oncology or from endoprosthetic hip-joint replacement surgeries at the Department of Orthopedic Surgery of the Medical Faculty Mannheim, Heidelberg University, Germany. Detailed clinical characteristics of patients and donors are provided in Table 1 and supplemental Table 1.

**Table 1.** **Cohort of patients for single-cell RNA sequencing of BM cells**

Sample ID	Origin	Age, sex	WHO 2017	Karyotype	Molecular genetics	% BM blasts	Previous treatment	Number of cells
HY1	Femur	30, M						651
HY2	Femur	65, M						1558
HY3	Femur	55, F						4190
HY4	Femur	70, F						1050
HY5	Femur	52, F						1576
HY6	Femur	90, F						2031
HY7	BM	72, F						524
MDS1	BM	81, M	MDS-MLD	46,XY [20]	*ASXL1*, *TET2*	<1%	Naive	481
MDS2	Femur	75, F	del5q	46,XX,del(5)(q13q31), inv(6)(p23q13)[3]/46,XX,del(5)(q13q31),der(11)t(11;17)(q23;p13) [3]	None	15%	Len, 5Aza	5422
MDS3	BM	79, M	del5q	46,XY,del(5)(q14q34) [19] 46,XY,del(5)(q14q34), t(7;9)(q21;q34)[3]	*TP53*	<5%	Len	1636
MDS4	Femur	86, M	MDS-MLD	46,XY,del(9)(q13q22) [11]	None	<5%	Naive	1348
MDS5	BM	78, M	MDS-MLD	46,XY[20]	*SF3B1*, *RUNX1*	<5%	5Aza	400
MDS6	BM	56, M	MDS-RS-T	46, XY, del (5)(q14), der(11)t(11;16) (q22;q12),der(16) t(11;16) (q23;p13) t(5;16)(q14;q12)[16]	*SF3B1*	<5%	Naive	418
MDS7	BM	67, F	MDS-MLD-RS	46,XX [20]	*SF3B1*	<5%	Naive	1383
MDS8	BM	83, M	MDS-MLD	46,XY[20]	*RUNX1*, BCOR	10%	5Aza	1703

5Aza, azacytidine; F, female; Len, lenalidomide; M, male; WHO, World Health Organization.

### Human specimen

Bone specimens were cut into small pieces, and bone fragments were washed 3 times with cooled phosphate-buffered saline + 4% fetal calf serum for 5 minutes and subsequently incubated with 2 mg/mL collagenase II (Sigma-Aldrich) for 25 minutes at 37°C. Subsequently, 40 μM filtered cells were subjected to erythrocyte lysis. We targeted for the analysis of 2 specimens from each patient (see supplemental Table 2): one unselected sample after collagenase digestion and a second sample enriched for stroma cells if the cell recovery after digestion was >10^6^ total viable cells. For the stroma sort, cells were blocked with human fragment crystallizable receptor (FcR) Blocking Reagent (Miltenyi Biotec) and stained with phycoerythrin (PE )CD45, PE CD3, PE CD19, PE CD11b, Allophycocyanin (APC) CD235a, PE-Cy7 CD71, and SYTOX Blue (Thermo Fisher Scientific) on ice and 0.01 μM Calcein AM (Thermo Fisher) for 20 minutes at room temperature. After washing, SYTOX Blue-negative, Calcein AM-positive, CD45^–^lin^–^CD235a^–^CD71^–^ cells were sorted using a BD FACSMelody (BD Bioscience). All samples (digested cells and sorted cells, if available) were additionally stained on ice with TotalSeq-A antibodies (BioLegend; supplemental Table 3) before library construction.

### Mouse experiments

All animal experiments were approved by the state authority in Karlsruhe, Germany. Xenotransplantation experiments were performed as previously described.^13^ In brief, 8-week-old female NOD scid gamma (NSG) mice (Jackson Laboratory, JAX stock #005557) were sublethally conditioned with busulfan (25 mg/kg) intraperitoneally, 48 and 24 hours^13^ before bilateral intrafemoral transplantation (IF TX of CD34^+^ hematopoietic stem cells (HSCs) and autologous mesenchymal stroma cells (MSCs) derived from patients with MDS and healthy (HY) donors (not overlapping with single-cell studied patients; supplemental Table 1).^14^^,^^15^ Long-term engraftment was assessed 12 weeks after transplant by BM aspiration, which was also performed in control mice who did not undergo transplantation. End points were conducted 24 to 29 weeks after IF TX. Conditioned control mice without human transplants were sacrificed at similar ages. At the end point, tibias, femurs, and hip bones were flushed to extract BM. Flushed bones were cut into pieces and digested with 2 mg/mL collagenase IV (Gibco) and 1 mg/mL dispase (Gibco) in 2 mL α-MEM + 10% fetal bovine serum 3 times for 10 minutes at 37°C. After erythrocyte lysis, human and murine CD45^+^ cells were depleted using MojoSortHuman CD45 Nanobeads (BioLegend) and mouse CD45 MicroBeads (Miltenyi Biotec) to enrich for the stromal compartment.

Isolated cells were blocked with human (h) and mouse (m) FcR Blocking Reagent (Miltenyi Biotec). Dead cells were excluded using SYTOX Blue (Thermo Fisher Scientific). BM from patient-derived xenograft (PDX) model was stained with hCD45 and mCD45 to determine human engraftment. Stromal cells were stained for PE hCD45, PE mLin, PE mCD19, PE mCD31, BV786 mCD51, PE mCD71, and APC mCD140a, and labeled with 0.01 μM Calcein AM (Biozol) for 20 minutes at room temperature. SYTOX Blue-negative, Calcein AM-positive, hCD45^–^mLin^–^mCD19^–^mCD31^–^mCD71^–^ cells were sorted and subjected to library construction. The negative cell fraction was further analyzed using mCD51 and mCD140a. Analysis and sorting were performed using a BD FACSMelody (BD Bioscience).

### Single-cell analysis library construction and analysis

In brief, single-cell libraries were generated using the Chromium Next GEM Single Cell 3 (v3.1) kit and custom, independent amplification for the CITE-seq libraires. Reads were processed using the CellRanger and Seurat pipeline. Interactions between cell populations were analyzed using CellPhoneDB and CrossTalkR. For a detailed description of the analytical process, see the supplemental Methods.

## Results

### Murine BM stroma is reprogrammed by exposure to human MDS xenografts to upregulate extracellular matrix (ECM) remodeling and HSC-supporting factors

**Figure 1.**
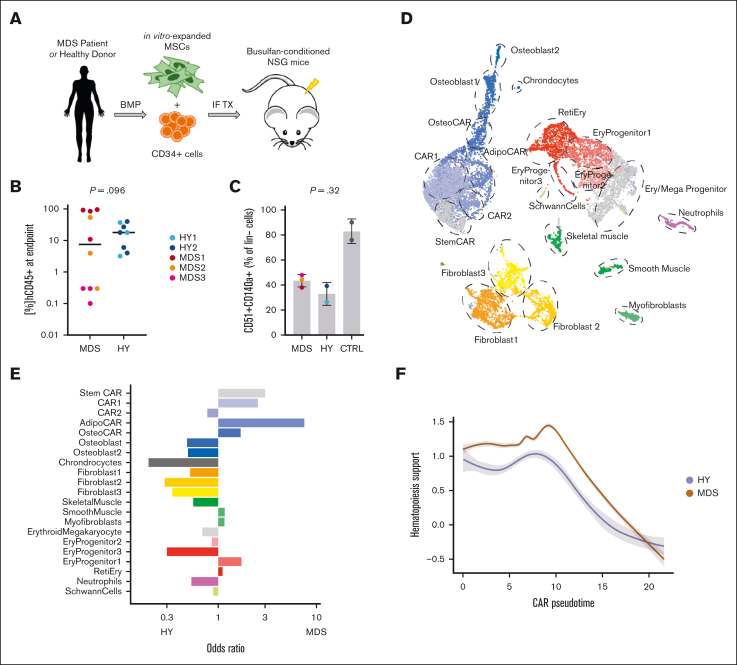
**MDS-derived changes in mice stromal compartment from xenograft transplantation experiments.** (A) Schematic workflow of transplantation setup: MDS- and HY-derived CD34^+^ cells were injected alongside in vitro expanded MSCs into NSG mice after busulfan conditioning. End point analysis was conducted at 24 to 29 weeks after transplant. (B) Quantified engraftment of n = 18 MDS and HY mice who underwent xenograft transplantation at end point; 2-sided *t* test. (C) Quantification of % CD51^+^CD140a^+^ double-positive cells from pooled mouse bones per sample donor as percent of murine lineage-negative cells. CTRL mice did not receive xenotransplantation; 2-sided *t* test. (D) UMAP clustering of n = 13 487 high-quality cells mapped to mouse (mm10) genome to identify stromal and residual hematopoietic cell populations. (E) Differential abundance of detected cell populations quantified as odds ratio for MDS vs HY sample origin. Positive ratio indicates increased abundance in MDS samples. (F) Gene expression of aggregated gene expression for Kitl, Il7, Igf1, Csf1, Bmp4, and Cxcl12 across CAR pseudotime differentiation. Gray shades represent standard error. CAR, Cxcl12^+^ abundant reticular; CTRL = Control; UMAP: Uniform Manifold Approximation and Projection; Ery, erythrocytes; IF TX, intrafemoral transplantation; Reti, reticulocytes.

We aimed to setup an experimental model that allowed us to analyze the changes in the BM stroma compartment in response to MDS hematopoiesis. Therefore, we established PDX that were either engrafted with primary human MDS or HY, age-matched human hematopoiesis, and focused our analysis on differential effects in the mouse stroma upon xenotransplantation. In total, n = 18 NSG mice were transplanted with CD34^+^ HSCs from n = 3 patients with MDS and n = 2 age-matched HY donors (Figure 1A; supplemental Table 1).^13^^,^^16^ Median long-term engraftment rates of human cells at the experimental end point 24 to 29 weeks after transplantation were 7.5% (range, 0.1%-95%) for MDS and 17.9% (range, 3.2%-40%) for HY samples (Figure 1B; supplemental Table 1). Positive engraftment was defined as >0.1% of hCD45^+^ cells of total hCD45^+^/mCD45^+^ cells (Figure 1B; supplemental Figure 1A). To discern induced effects in the BM stroma, we pooled bones from all mice per patient, performed collagenase digestion, and used fluorescence-activated cell sorter (FACS) to enrich for the nonhematopoietic cell population (supplemental Figure 1B). Within this lineage-negative population, we quantified the fraction of murine MSCs with the canonical markers CD140a (*Pdgfra*) and CD51 by flow cytometry. We observed that mice engrafted with MDS hematopoiesis had a slightly increased abundance of CD140a^+^CD51^+^ cells as compared with HY controls (43% vs 33%; Figure 1C). However, those populations were overall reduced in conditioned but not controls who received xenotransplantation. To explore the murine MSC population of cells after human MDS or HY HSC exposure more closely using single-cell sequencing analyses, we sorted 5000 SYTOX Blue-negative, Calcein AM-positive, and mLin^–^ cells from each patient with MDS and HY donors specific murine-enriched MSC cell pool. In total, we recovered 13 487 cells after quality controls comprised n = 15 different niche cell populations (Figure 1D; supplemental Figure 1C-D).

Within these populations, MSCs expressing high levels of Cxcl12 were most abundant and are therefore referred to as *Cxcl12*^+^ abundant reticular chimeric antigen receptor (CAR) cells hereafter. CAR cells could be further subdivided into distinct populations and showed major differentiation trajectories. Most prominent were the CAR with osteogenic priming (OsteoCAR) population (*Cxcl12*^*+*^*Alpl*^+^) and more differentiated osteoblasts (*Col1a1*^*+*^*Bglap*^*+*^), chondrocytes (*Col2a1*^+^), and CARs primed to adipogenic differentiation (*Lepr*) (supplemental Table 4). We further grouped 3 primitive CAR populations as StemCAR (lowest pseudotime) and 2 primitive CAR populations 1 and 2, which showed differences in marker gene expression such as *Cxcl12* and *Kit* (supplemental Figure 2). Interestingly, these prominent subpopulations were also differentially represented in the niche of MDS and HY xenografts, that is, CAR1, CAR2, and AdipoCARs were more prevalent in patients with MDS than in HY donors. Whereas osteoblastic and fibroblastic niche cell populations, as distinguished by the expression of *Cd34*, *Htra4/Col22a1*, and *Fmod/Fibin*, were reduced in MDS as compared with HY donors (Figure 1E). As reported previously,^17^ our applied sorting approach also yielded Schwann cells featuring *Mag* expression, and 3 populations of muscle cells characterized as smooth muscle cells (*Acta2*), myofibroblasts, and skeletal muscle cells (*Acta1*), most likely derived from surrounding soft tissue. We also detected small amounts of residual hematopoietic cells that mostly belonged to the erythropoietic lineage, which probably passed through the lineage depletion process because of their low CD45 expression during erythropoietic differentiation. To address this more closely, we performed differential gene expression analysis of these specific BM cell populations to compare the effects of exposure to either MDS or HY hematopoiesis (supplemental Table 5; supplemental Figure 3).

All CAR cell populations were most affected by MDS hematopoiesis and showed a higher number of differentially expressed genes than other subpopulations (supplemental Figure 3A). Notably, the CAR2 population, which was least affected by differential abundance, showed the highest number of differentially expressed genes (supplemental Figure 3B; supplemental Table 5), including numerous key players of ECM reorganization and hematopoietic supporting factors, such as *Cxcl12*, *Il7*, *Lox*, and *Loxl1*, among many others. To more broadly quantify this hematopoiesis-supporting gene expression pattern of stromal cells, we aggregated gene programs of hematopoiesis-supporting factors (*Cxcl12*, *Kitl*, *Il7*, and *Igf1*) and observed a significant increase in this gene expression signature in the most primitive CAR cells upon engraftment with MDS than in those with HY (Figure 1F). Although there was no correlation between the expression levels of these hematopoiesis-supporting genes and the total human engraftment as a functional readout, they correlated with myeloid engraftment in xenografts (supplemental Figure 4), which supports the notion that stromal cell gene expression and malignant MDS hematopoiesis are correlated in a myeloid supportive state and may drive ECM remodeling.

In summary, our data indicated that upon exposure to MDS hematopoiesis, the relative abundance of CAR cells expanded, which is known to be critical to maintaining stem cell function.

### Establishment of a BM niche cell–enriched single-cell atlas of HY and MDS human BM

**Figure 2.**
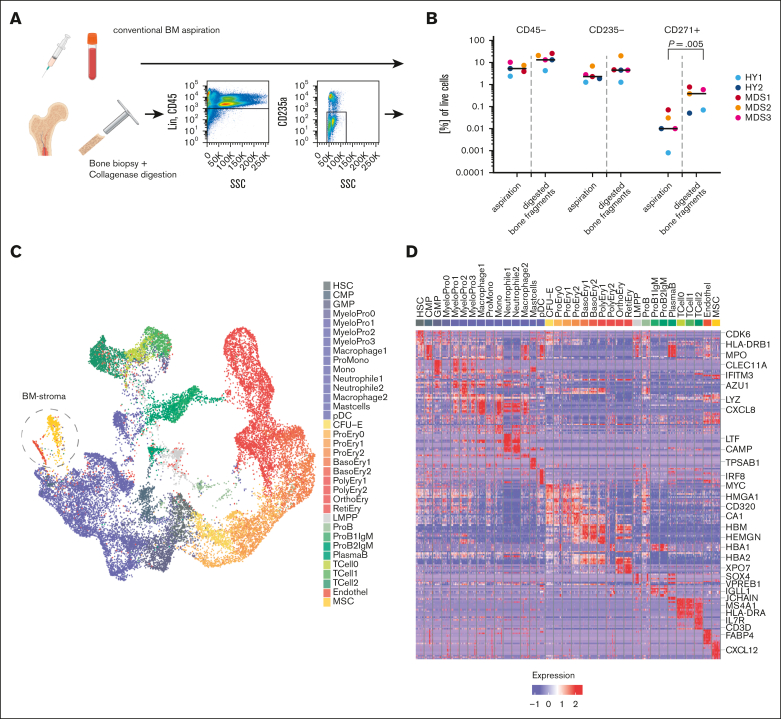
**Single-cell landscape of primary BM from patients with MDS.** (A) Schema of the experimental workflow for primary human cells: liquid BM aspirates and BM trephines derived from diagnostic BM punctures and hip replacement surgeries were digested or sorted for SYTOX Blue-negative Calcein AM-positive lin^–^CD45^–^CD235a^–^ cells and subjected to 10x single-cell RNA sequencing. (B) Flow cytometric quantification of the proportion of CD45^–^, CD235a^–^, and CD45^–^CD235a^–^CD271^+^ populations for n = 5 paired samples of BM aspirations and digested trephines; paired, 2-sided *t* test. (C) UMAP plot of 24 371 high-quality primary cells comprising n = 35 color-coded distinct cell populations. (D) Heat map of top 10 marker genes from 100 randomly sampled cells per population; see also supplemental Table 6 and selected genes highlighted. Figure 2A created with BioRender.com.

We next sought to translate our findings from the PDX experiments to primary samples from patients with MDS. Because obtaining naive BM niche cells in primary tissue is challenging, we developed an experimental setup aiming for an unbiased sampling of the primary BM and to further address rare niche and nonhematopoietic cell populations (Figure 2A). Besides conventional BM aspirations, intact bone samples were obtained from 7 aged HY donors and 8 patients with MDS who had either undergone hip replacement surgery or had BM trephines collected for diagnostic purposes (Table 1). To increase the yield of nonhematopoietic cells, these bone fragments were digested with collagenase. We then sought to analyze 2 samples per patient: first, all recovered cells after digestion, and a second subsequent sample enriched by FACS for SYTOX Blue-negative Calcein AM-positive lin^–^CD45^–^CD235a^–^ cells for samples with sufficient yield (supplemental Figure 5A; supplemental Table 2). This strategy increased the frequency of stromal cells by >10-fold compared with their extraction from a standard BM aspiration (Figure 2A-B) while preserving the immunophenotyped of MSC (supplemental Figure 5B).

With this approach, we captured a median of 1365 cells per patient, adding up to a total of 24 371 primary cells to enable the transcriptomic dissection of the human BM of HY donors and patients with MDS on a single-cell level, including the nonhematopoietic MSC and endothelial compartment (Figure 2C; supplemental Figure 6A). This data set clustered into n = 35 distinct transcriptional profiles (Figure 2D; supplemental Table 6) and subdivided various states of hematopoietic differentiation. In addition, this was confirmed by pseudotime analysis outlining all major hematopoietic differentiation routes (supplemental Figure 6B).

### Comparison of HY and MDS hematopoiesis on single-cell level reveals reduced differentiation, increased inflammatory IL-1 signatures, and increased cyclin dependent kinase 6 expression in MDS

**Figure 3.**
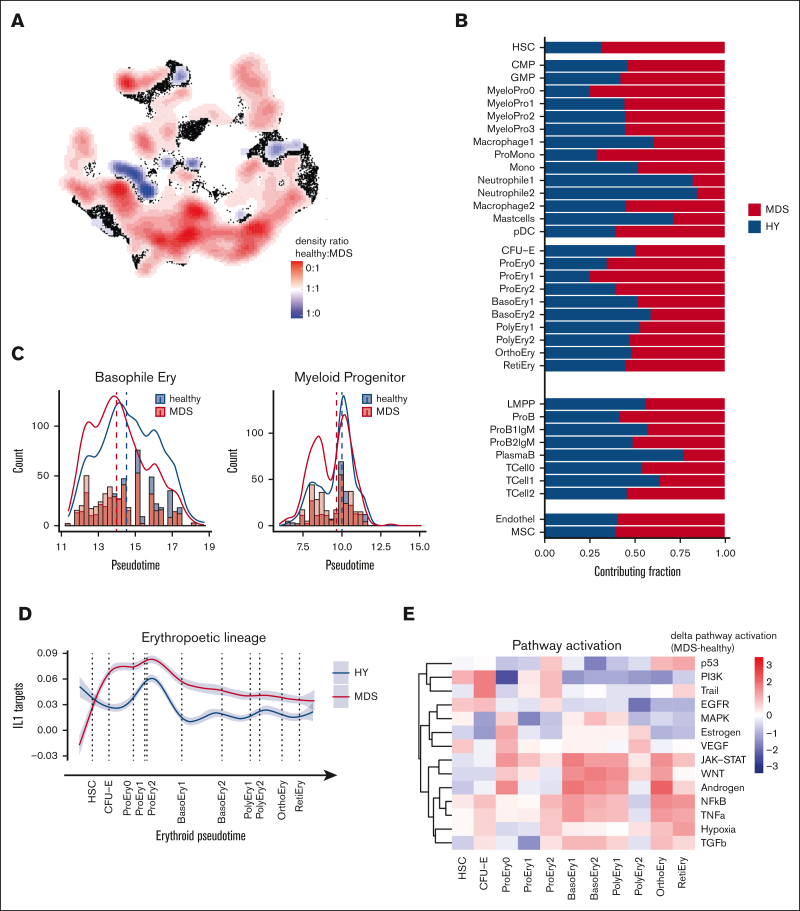

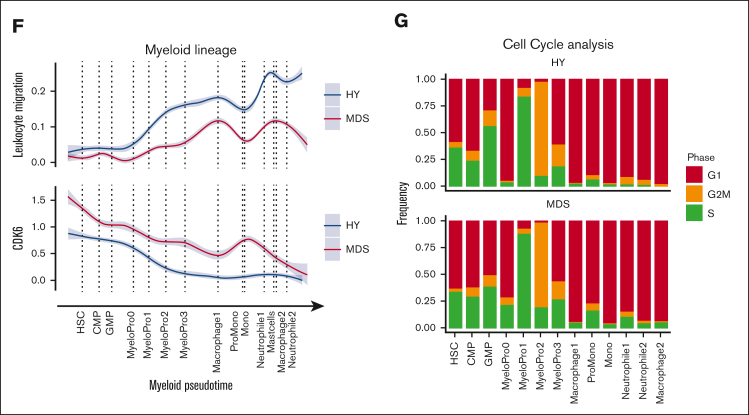
**Differential abundance and gene expression in primary MDS cells.** (A) Primary cells in UMAP representation overlaid with density plot for differential abundance in MDS vs HY cohort (see also supplemental Figures 5A and 6). (B) Barplot of frequency per cell population for n = 11 580 HY and 12 791 MDS cells. (C) Density histogram of pseudotime estimate for n = 2 selected cell populations separated by sample origin. Dashed lines indicate the median per cell population in condition, 2-sided *t* test adjusted using Benjamini-Hochberg procedure. (D) Gene expression along erythroid pseudotime differentiation for subset of n = 9168 cells designated to erythropoiesis of averaged gene expression using (AddModuleScore) for IL1 target genes according to gene ontology (GO:0070498). Gray shades represent standard error. (E) Progeny analysis for pathway activation in erythropoietic cells. (F) Gene expression along myeloid pseudotime differentiation for subset of n = 9064 cells designated to myelopoiesis, an averaged gene expression using for leucocyte migration–related genes according Kyoto Encyclopedia of Genes and Genomes (KEGG) pathway (hsa04670, top), and cyclin dependent kinase 6, (bottom). (G) Cell cycle analysis derived from transcriptomic data for myeloid progenitor cells groups per cell population. CMP, common myleoid progenitor; GMP, granulocyte/monocyte progenitor.

Based on the comprehensive single-cell transcriptomics atlas of HY- and MDS-derived BM, we next aimed to identify deregulated pathways in MDS as compared with HY. Thereby, we identified the MDS-typical abnormal distribution of differentiated stages of hematopoietic progenitor cells (Figure 3A). In particular, we observed a decrease of mature neutrophil cell populations in MDS (neutrophils 1/2, 388 vs 84 and 463 vs 85; 82% and 84% HY; adjusted *P* [*P*adj] = 1E-54, 1E-72; Figure 3B) and an accumulation of immature myeloid cells (25% HY; *P*adj = 7E-45; MyeloPro0) and promonocytes (ProMono, 28% HY; *P*adj = 1E-42; Figure 3B; supplemental Table 7). Beyond these differential abundances, we interrogated the distribution of pseudotime analysis as an estimate of hematopoietic differentiation and observed notable differences between HY and MDS. In this analysis, cells classified as intermediate erythropoietic progenitors (basophile erythropoietic progenitors) showed an overall decreased pseudotime estimate for n = 415 MDS and n = 594 HY cells (*P*adj = 9E-8; Figure 3C). Similarly, myeloid progenitors from patients with MDS showed skewed differentiation estimates to more earlier pseudotime estimates (*P*adj < 3E-5; Figure 3C). These marked differences were also reflected in the altered transcriptional programs during hematopoietic differentiation. As an example, in erythropoietic cells, we noted an increased IL1 signature most prominent in early erythropoietic MDS cells (Figure 3D). The same IL-1 signature was also overexpressed in murine PDX-derived erythropoietic cells (supplemental Figure 7) and was evident by pathway activation analysis using a computational approach to extract signaling pathway activation (PROGENy).^18^ This analysis showed an increased activity of proinflammatory and proangiogenic pathways in MDS, such as tumor necrosis factor-alpha, as well as the JAK-STAT and WNT pathways in intermediate erythropoietic differentiation (Figure 3E). Across myeloid differentiation, we observed profound alterations in the timely ordered expression of chemotaxis and leukocyte adhesion programs (Figure 3F) as well as increased expression of cyclin dependent kinase 6 in MDS, suggesting increased cell cycle activation (Figure 3F). This was corroborated by an increased proportion of potential blast-like cells in S/G2 phase expressing these gene patterns, for example, MyeloPro0 in S phase (4% in HY vs 28% in MDS, Figure 3G). In summary, these findings highlighted far-reaching alterations across all hematopoietic states in MDS compared with aged HY hematopoiesis.

### Rare primary mesenchymal cells are reprogrammed to increased hematopoietic support in a patient-specific manner

**Figure 4.**
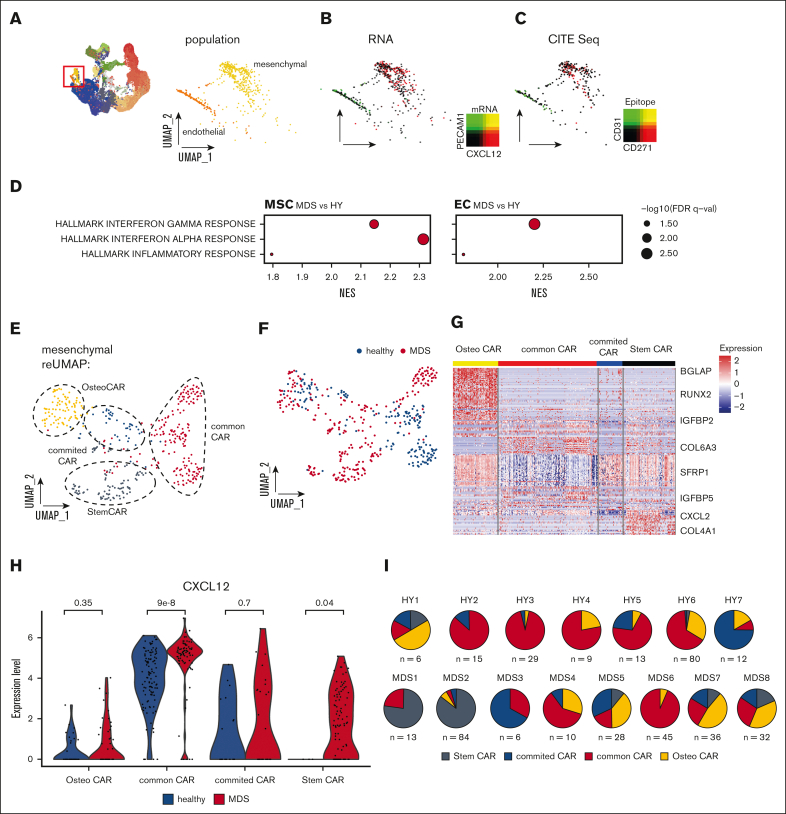
**Primary mesenchymal niche in MDS.** (A) UMAP overview of total cell populations with highlighted cells of nonhematopoietic origin, magnified and extracted in right panel. (B) Gene expression highlighted in UMAP subspace for the key mesenchymal marker CXCL12 and endothelial marker PECAM1. (C) Epitope density derived from CITE-seq for CD271 and CD31, 2-sided *t* test. (D) Differential gene expression between pseudobulked MSCs and ECs for MDS/HY comparison. (E-F) Subclustering of n = 418 primary classified MSCs resembles 6 subpopulations that were color-coded. (G) Heat map of marker genes for different MSCs subpopulations, z scored. (H) Gene expression of CXCL12 per MSC subcluster and condition; each dot represents a CXCL12-expressing cell. (I) Quantification of distribution of MSC subpopulations for each donor. FDR, adjusted false discovery rate; NES, normalized enrichment score.

After the enrichment of BM niche cells in our samples, we were especially interested in exploring cells of nonhematopoietic origin in our data set. With our experimental and analytical pipeline, we detected 2 nonhematopoietic cell populations that we designated as endothelial cells (ECs) and MSCs (Figure 4A). n = 163 cells (total 0.7%, 0.5% in HY, and 0.7% in MDS) were classified as ECs by expression of key markers such as Fatty Acid Binding Protein 4(FABP4), von Willebrand factor, and Platelet And Endothelial Cell Adhesion Molecule 1(PECAM1) (supplemental Table 6). MSCs expressing CXCL12, Insulin-like growth factor-binding protein 5 (IGFBP5), or collagen type I alpha 2 comprised a total of n = 418 cells (total 1.7%, 1.4% in HY, and 1.9% in MDS; Figure 4B). Furthermore, we validated the stroma affiliation of these cell types by epitope quantification with CITE-seq, which demonstrated that these 2 cell types mutually exclusively expressed CD31 and CD271 (Figure 4C). In a global gene expression comparison between MDS- and HY-derived MSCs and ECs, both cell populations showed a strong upregulation of inflammatory gene sets (Figure 4D; supplemental Figure 8). To more granularly analyze the mesenchymal cells, we conducted a subcluster analysis of n = 418 MSCs that uncovered similar subpopulations as in the PDX experiments. Based on differential expression and pseudotime, we grouped 4 distinct subpopulations, which were separable by differential expression of CXCL12, BGLAP, Leukemia inhibitory factor receptor (LIFR), and IGFBP4 (Figure 4E-G). These mesenchymal populations likely resemble bona fide mesenchymal stem cells (lowest pseudotime estimate, StemCAR), common CAR (without evident differentiation priming), CAR with osteogenic priming (OsteoCAR, high BGLAP/Osteocalcin expression), and CAR with intermediate differentiation state (committed CARs, (Figure 4F; supplemental Figure 9A).

Given the increased secretory and hematopoietic supporting phenotype in MSCs exposed to MDS hematopoiesis in our experimental PDX model (Figure 1D), we were also specifically interested in altered mesenchymal function in primary MDS BM. To address this, given the strong heterogeneity among the primary patient samples observed above, we proceeded by analyzing the hematopoietic support in individual subclusters. We assessed the expression of CXCL12 and other canonical factors of hematopoietic support, such as Kit Ligand(KITL) and IL7 (supplemental Table 8). We found that especially in the common MSC populations, but also StemCAR strongly overexpressed CXCL12 (Figure 4H) as well as an independent gene expression program derived from unbiased consensus non-negative matrix factorization (supplemental Figure 9B-C).^19^ Further, StemCAR cells were almost exclusively found in MDS (*P* < 2.2e-16; Figure 4H). In addition, to address whether the accumulation of immature MDS cells is linked with high expression of CXCL12, we quantified the expression in ex vivo expanded MSCs from n = 6 high-risk patients with MDS, which achieved complete remission (ie, blast clearance <5%) upon treatment with hypomethylating agents, and observed that the MSCs at remission significantly expressed lower levels of CXCL12 (supplemental Figure 9D).

This suggests that this altered specialized secretory mesenchymal state is only present in a subset of patients with MDS and may support the malignant hematopoietic clones by overexpressing key hematopoietic factors.

### Interactome-wide analyses identify novel signaling axes with BM niche cells

Given that specialized MSC populations only contribute to <1% of the BM cellularity but may influence multiple populations with signaling molecules, we aimed to annotate potentially more globally disrupted signaling patterns and interactomes in the human MDS BM between all cells. Therefore, we applied the CellPhoneDB^20^ and RNAMagnet^21^ tools, which use curated databases of receptors, ligands, and their interactions for inference of cellular communication. To maximize the yield on interaction patterns, we further extended these ligand-receptor databases by omnipath^22^ (as reported by Leimkühler et at^17^), specifically adding data about signaling pathways in the BM.

**Figure 5.**
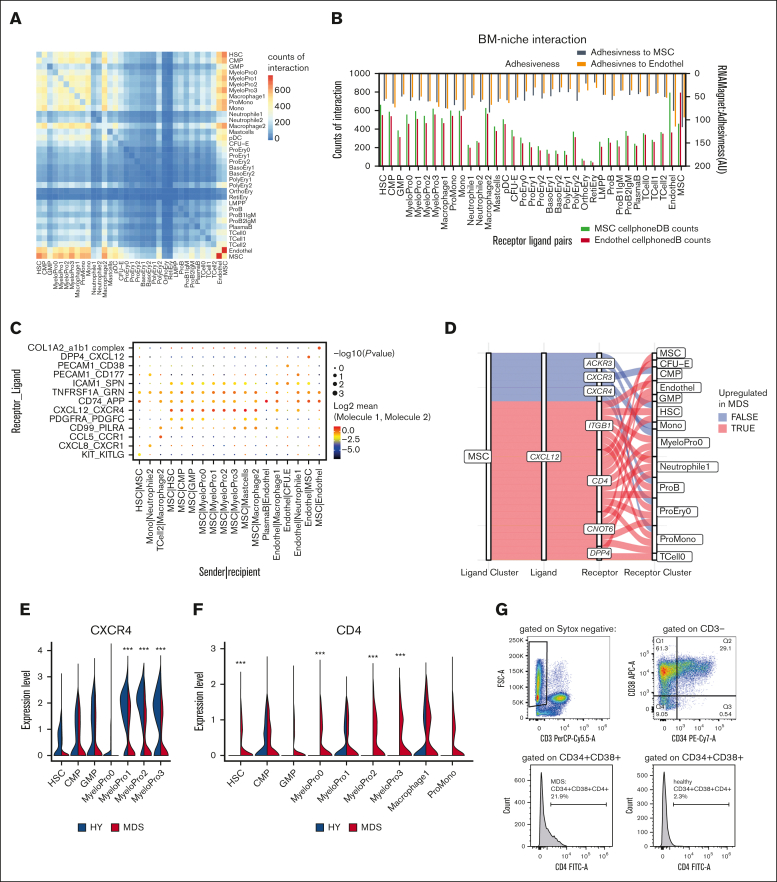
**Interactome in primary MDS BM.** (A) Heat map of number of pairwise expressed genes for interacting receptor-ligand pairs as quantified by CellPhoneDB. (B) Interaction of hematopoietic cells with MSCs and ECs quantified by number of expressed receptor-ligand pairs (bottom scale) and adhesiveness (top scale) in arbitrary units (AU) derived from RNAMagnet. (C) Exemplary quantitative expression of key receptor-ligand pairs for stromal-hematopoietic interaction for selected cell pairs. (D) Sankey plot for CXCL12 signaling from MSCs with various receptors to hematopoietic cells. Color-coded whether a specific ligand-receptor axis between cell populations is differentially active in MDS over HY. (E-F) Quantitative gene expression of CXCR4 and CD4 in myeloid progenitor cells in HY and MDS comparison, 2-sided Wilcox test derived from FindMarker function. (G) Quantification of CD4 expression in myeloid progenitor cells as gated by CD34^+^CD38^+^.

Hereby, a matrix of each permutation between the detected cell types was calculated (Figure 5A). This revealed that ECs and MSCs had the highest interaction count with each other, indicated by the highest number of expressed ligand-receptor pairs, followed by interaction with immature myeloid cell populations (Figure 5A). To validate this approach, we further applied RNAMagnet to infer adhesiveness and cellular contact^21^ (Figure 5B), which correlated well with the CellPhoneDB approach (r = 0.72; *P* < .0001; supplemental Figure 10A-B).

With both approaches, we found that most ligand-to-receptor interactions between MSCs and ECs (n = 232) consisted of collagen- and integrin-based interactions, such as the collagen type I alpha 2 and integrin complex (a1b1 complex; Figure 5C). Further, we noticed that HSCs and early progenitors expressed a similar number of ligand-to-receptor interactions with MSCs and ECs (for HSCs, n = 661 to MSCs and n = 550 to ECs) as compared with monocytes and macrophages (eg, macrophage1, n = 517 to MSCs and n = 544 to ECs; Figure 5B). Notably, cells with progressive erythroid differentiation expressed fewer interacting receptor-ligand pairs. Reticulocytes expressed only n = 56 interacting gene pairs with MSCs and n = 39 with ECs, possibly reflective of maturation and subsequent liberation into the peripheral blood.

Such ligand-to-receptor expression was highly specific for certain cell types (Figure 5C). For instance, KIT and KITLG were exclusively exchanged between HSCs and MSCs. For CXCL12, various signaling pathways appeared in this data set: Dipeptidylpeptidase 4 (DPP4) was exclusively expressed on ECs and is known for interaction with CXCL12, suggesting a nonhematopoietic axis of CXCL12 signaling in the BM. In contrast, CXCR4, the canonical receptor for stroma-derived CXCL12, was expressed in early myeloid progenitor cells (Figure 5C).

To interrogate quantitative differences in this interactome of MDS, we next quantified the expression patterns separately for MDS and HY and calculated differential estimates using CrossTalkeR^23^ across all cellular interactions, also including signaling within hematopoietic cells. This revealed a magnitude of differential interactions between cell types (supplemental Table 9).

CXCL12, which we found to be overexpressed in IGFB4^high^ MDS-MSC, showed multiple signaling pathways through multiple receptors (Figure 5D; eg, CXCR4, ITGB1, and CD4) expressed from various cell populations in the BM. The canonical CXCL12-CXCR4 axis was decreased in MDS stem and progenitor cells because of decreased receptor expression in various myeloid progenitor populations (Figure 5E). Instead, we observed an aberrant CD4 expression on myeloid progenitor cells that also acts as a receptor for CXCL12 (Figure 5F). This aberrant expression was validated in CD34^+^CD38^+^ cells from the same patients with MDS (Figure 5G).

In conclusion, we provide insights into the interaction of MSCs with various hematopoietic compartments, highlighting that stromal cells alter their cellular profile and secretory behavior in response to MDS hematopoiesis, and present a data set that sheds light on the aberrant intercellular communication in the BM of patients with MDS.

## Discussion

Here, we describe a comprehensive single-cell atlas of the human and murine BM that highlights multilayered aberrant phenotypic changes in stromal cells in response to MDS hematopoiesis. To study the induced effects of primary engrafted MDS samples, coinjection of human ex vivo expanded MSCs was used to improve engraftment, as done by many other groups.^14^^,^^16^^,^^24^ Although this may confound the stroma in the murine BM niche directly after transplantation, these coinjected MSCs were shown to be cleared within 2 weeks of engraftment in previous studies.^14^^,^^16^^,^^24^ This argues that the induced effects on the murine stroma after 4 months of engraftment are an effect not mediated by human MSCs but of the remaining, long-term engrafted human hematopoietic cells. Nevertheless, we cannot fully exclude the possibility that differences between MSCs from HY controls and patients with MDS may imprint a long-lasting change within the endogenous mouse MSC niche. The MSCs identified in this study are in line with lineage-negative^11^^,^^21^ or LepR-positive selection studies before,^12^^,^^25^ but represent a much greater heterogeneity in the human data set that has not been reported before for the stromal population yet, but mimics the heterogeneity of AML blast cells published previously.^26^^,^^27^

We infer the lineage trajectories of MSCs from gene expression profiling, which, especially in the xenograft experiments, highlight a noncell autonomous role of this differentiation priming. The biased lineage priming is in line with previous reports that show increased adipogenic differentiation potential^28^^,^^29^ of primary MDS-derived MSCs to the expense of decreased osteogenic.^30^ Hayashi and colleagues^25^ also reported decreased osteogenic differentiation in stromal cells upon contact with MDS hematopoiesis in a syngeneic mouse model of MDS, which supports the finding that hematopoiesis affects differentiation in MSCs. These previous studies derived from FACS-purified primary MSCs from patients with MDS^4^^,^^31^ also highlight the inflammatory-primed state of the MSCs. We observed inflammatory gene expression profiles in both stroma and erythropoietic lineages and show that these are further present in ECs. Interestingly, this was also present in not clonally affected mouse erythropoietic cells, which suggests a noncell autonomous role for such altered gene expression patterns. Particularly, such inflammatory conditions driven by stroma-secreted alarmins have been found to contribute to chemoresistance in AML.^32^ We also found that MSCs directly support MDS cells by overexpression, for example, CXCL12, which is in line with findings in late-stage myelofibrosis^17^ and demonstrates that other leukemic cells may also benefit from such extrinsic growth stimuli.^11^^,^^33^ Whether our observed overexpression of such markers eventually also correlates with long-lasting remodeling of the BM niche needs to be further exported by spatial studies addressing bone architecture.^34^^,^^35^

Our study comprises a large atlas of studied cells but remains limited by the intrinsic heterogeneity of MDS, the small number of recovered stromal cells and patients’ studies, and ongoing difficulties to uniformly engraft primary low-risk MDS. In contrast, the systematic collection of intact BM fragments has never been reported in such detail. The presented data set provides an atlas of the cellular interaction of hematopoietic cells with the complete complexity of the BM, representing a valuable resource to further study intercellular signaling. Exemplary routes are the stromal CXCL12-CD4 axis between human macrophages or natural killer cells, which might be species specific.^36^ As such, CD4 has been shown to play a role in differentiation, activation, cytokine expression, and cell migration in myeloid cells in a T-cell receptor–independent pathway.^37^ In the future, such routes might become promising targets for further therapeutic developments and can be used independent of clonally altered hematopoietic cells, which may provide a more universal approach.

Conflict-of-intreset disclosure: The authors declare no competing financial interests.
