# Supplementary material for: Instructive interaction between myelodysplastic hematopoiesis and the bone marrow microenvironment at the single-cell level
Source: Blood Neoplasia. 2024 May 21;1(3):100021. doi: 10.1016/j.bneo.2024.100021 (PMC12082128; doi:10.1016/j.bneo.2024.100021)

Supplemental Figure 1:

A) Exemplary flow cytometry plot quantifying human engraftment of SYTOX Blue- cells for murine CD45 (mCD45) and human CD45 (hCD45). **B)** Gating strategy to assess mouse MSCs. **C)** UMAP plot of isolated bone marrow niche cells after engraftment with MDS or HY hematopoiesis colored by sample origin. **D)** Heatmap of top 100 marker genes from 100 randomly sampled cells per population for mouse dataset, z scored.

Supplemental Figure 2:

A) Violin Plots for selected to differential genes between CAR1 and CAR2. **B)** Boxplot of pseudotime estimate for CAR populations. **C)** Heatmap of differential expressed genes between CAR1 and 2.

Supplemental Figure 3:

A) Correlation of differentially expressed genes called by Seurat FindMarkers and absolute cell number of the shown populations captured in the scRNA dataset. **B)** Volcano plot of differentially expressed genes in CAR2 population. Genes with adjusted p values < 0.01 and log fold change > 0.5 are highlighted in red. Genes with biological impact, which were significantly differently regulated in more than n=2 comparisons, were annotated in the text. See also **Supplemental Table 5**.

Supplemental Figure 4:

A) Correlation of human engraftment (hCD45, CD33, CD34) in xenografts with aggregated gene expression for Kitl, Il7, Igf1, Csf1, Bmp4 and Cxcl12 per patient **B)** Correlation of human engraftment (hCD45, CD33, CD34) with injected number of CD34+ cells. Pearson correlation and p value.

Supplemental Figure 5:

A) Complete primary human dataset separated by sample processing in UMAP representation. **B)** Histogram of FACS derived expression for MSCs surface markers for ex vivo expanded MSCs from BM trephine and aspiration for a matched patient sample.

Supplemental Figure 6:

A) Human cells in UMAP scape separately plotted by donor origin. **B)** Pseudotime estimate projected onto the UMAP.

Supplemental Figure 7:

Boxplot of mouse IL1 target genes according to gene ontology (GO:0070498) for captured murine erythropoietic cells. Two sided t.test.

Supplemental Figure 8:

Correlation of aggregated gene expression (AddModule) for Hallmark Interferon alpha and IL1 target genes for human dataset separated by cell population and sample origin.

Supplemental Figure 9:

A) Expression of the four marker genes CXCL12, BGLAP (Bone Gamma-Carboxyglutamate Protein / Osteocalcin), IGFBP4 (Insulin Like Growth Factor Binding Protein 4) and LIFR (LIF Receptor Subunit Alpha) and pseudotime in MSC UMAP space. **B)** Heatmap of cNMF derived gene programs for MSC populations clustered. Right: z scored contribution of genes to hematopoietic support program. **C)** Gene expression of hematopoietic support program per MSC subcluster and condition, two sided t.test. **D)** Gene expression of CXCL12 normalized to HPRT1 in ex vivo expanded MSCs from MDS patients at best clinical response to hypomethylating agents (HMA) treatment, one-sided p value.

Supplemental Figure 10:

A) Boxplot of RNAmagnet derived adhesiveness for each cell population and sample origin. **B)** Scatter plot of RNAmagnet derived adhesiveness (score of the strength of attraction) and cellphoneDB counts (number of expressed receptor:ligand pairs). Each dot represents an interaction between two cell populations.

Supplemental Figure 1

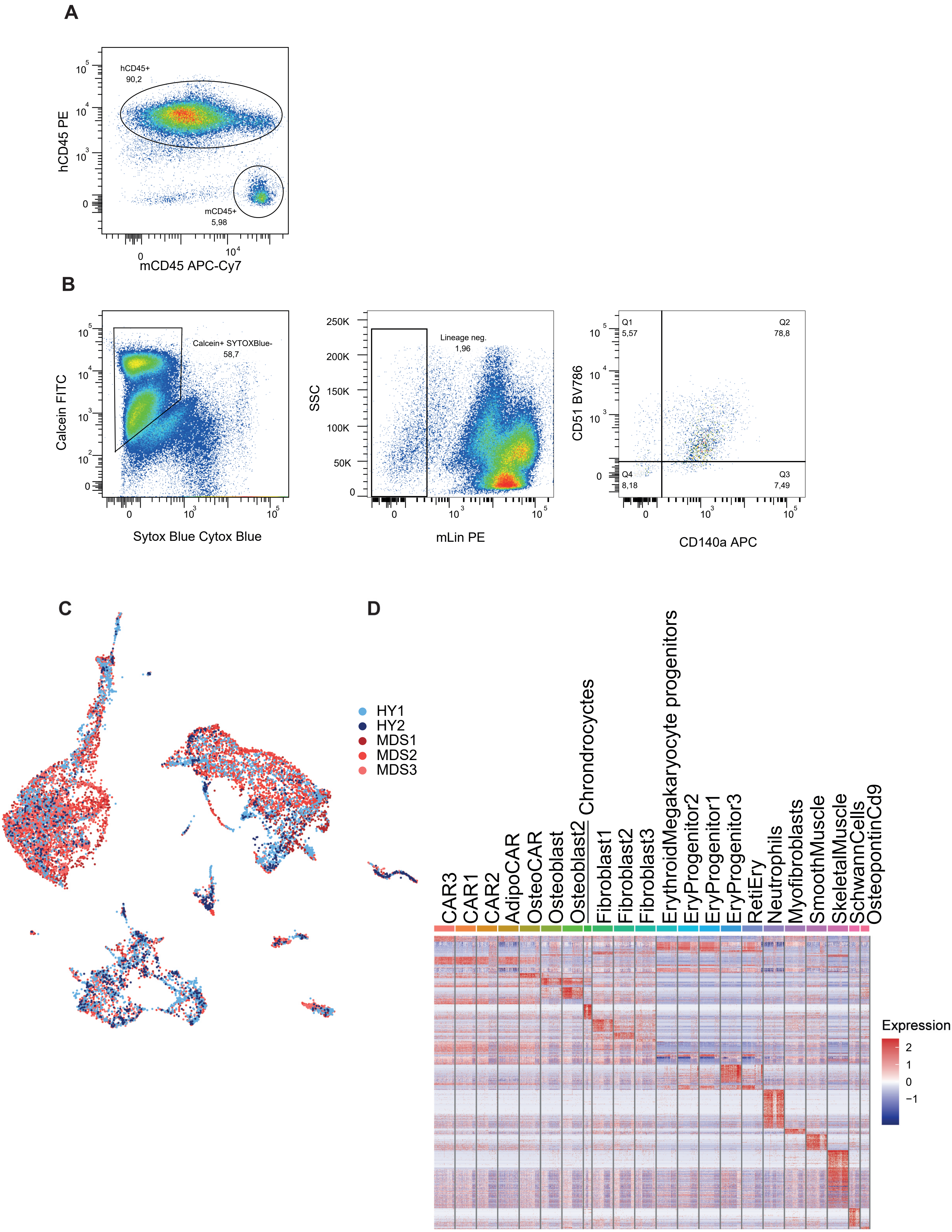

Supplemental Figure 2

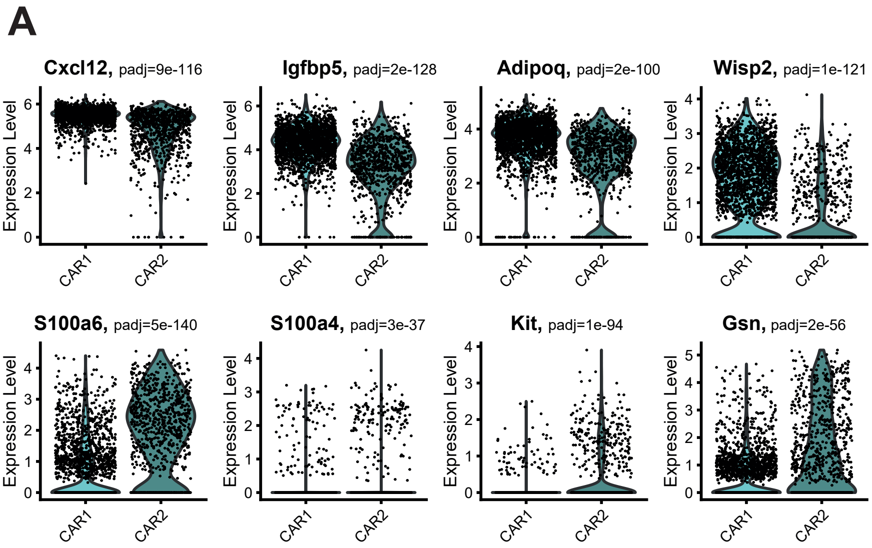

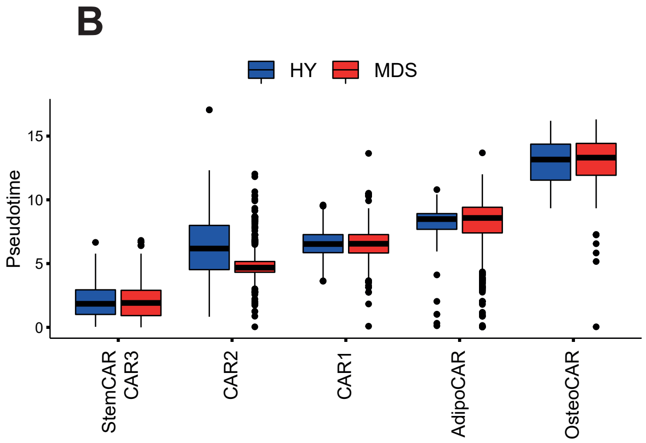

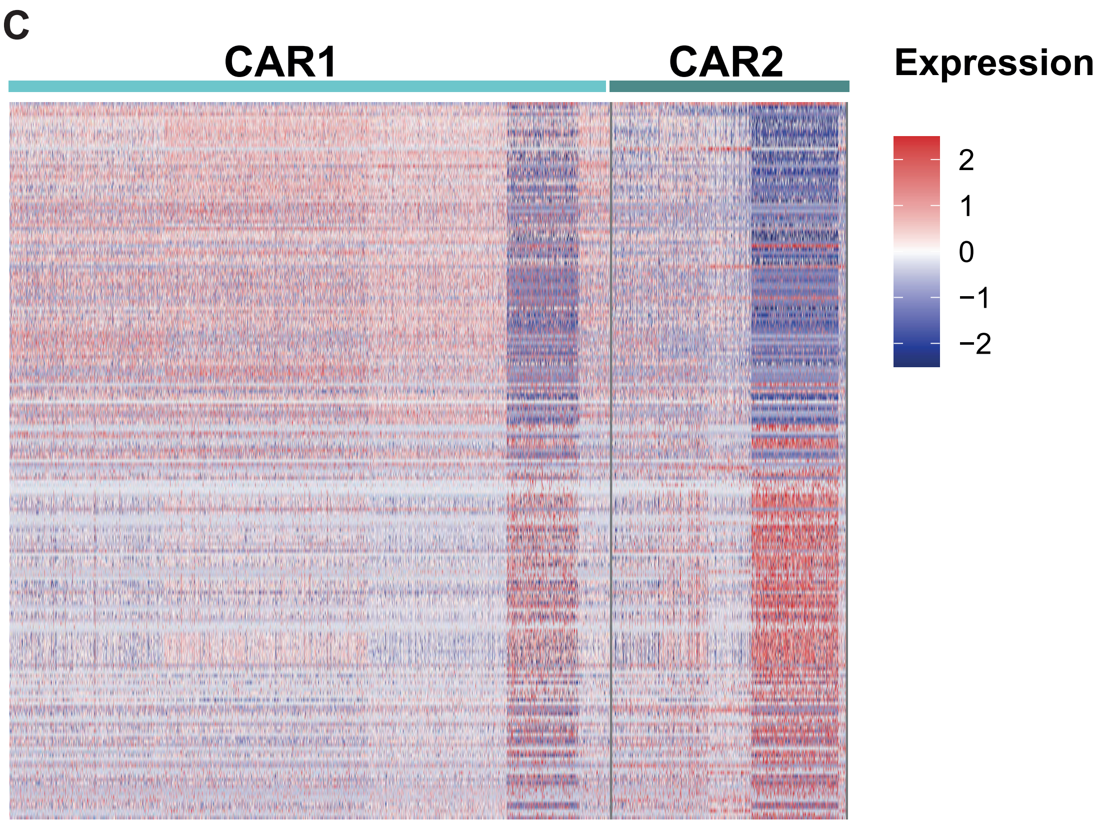

Supplemental Figure 3

A

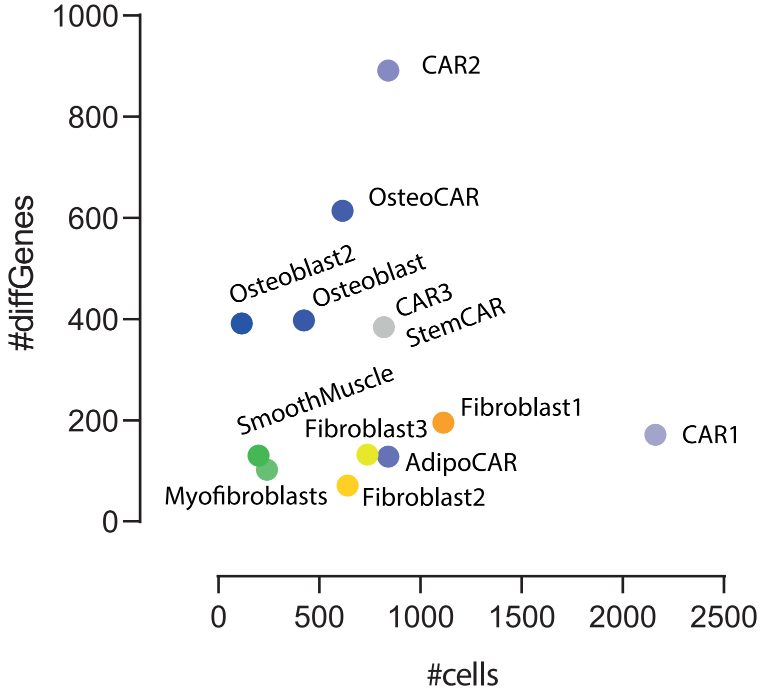

B

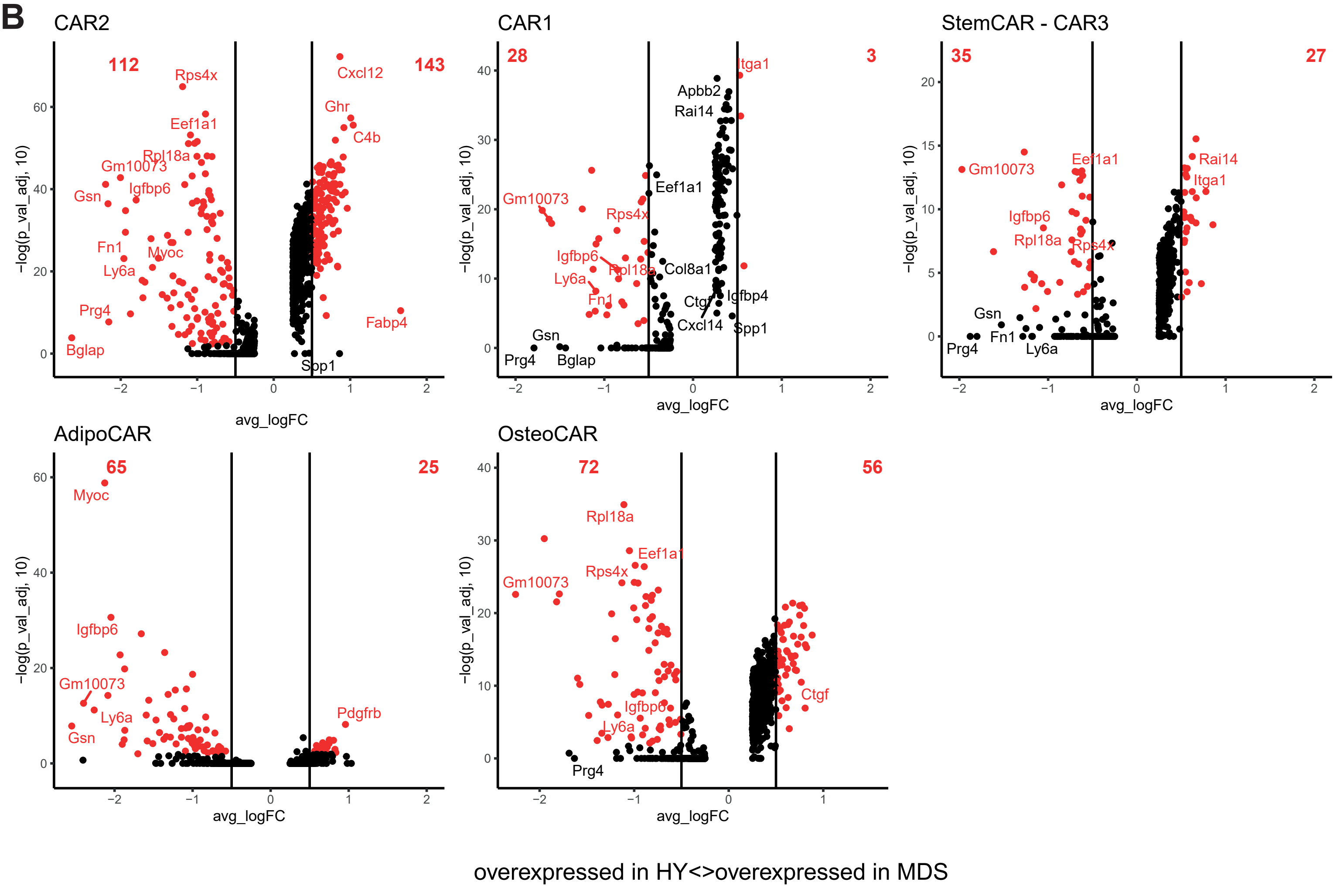

Supplemental Figure 4

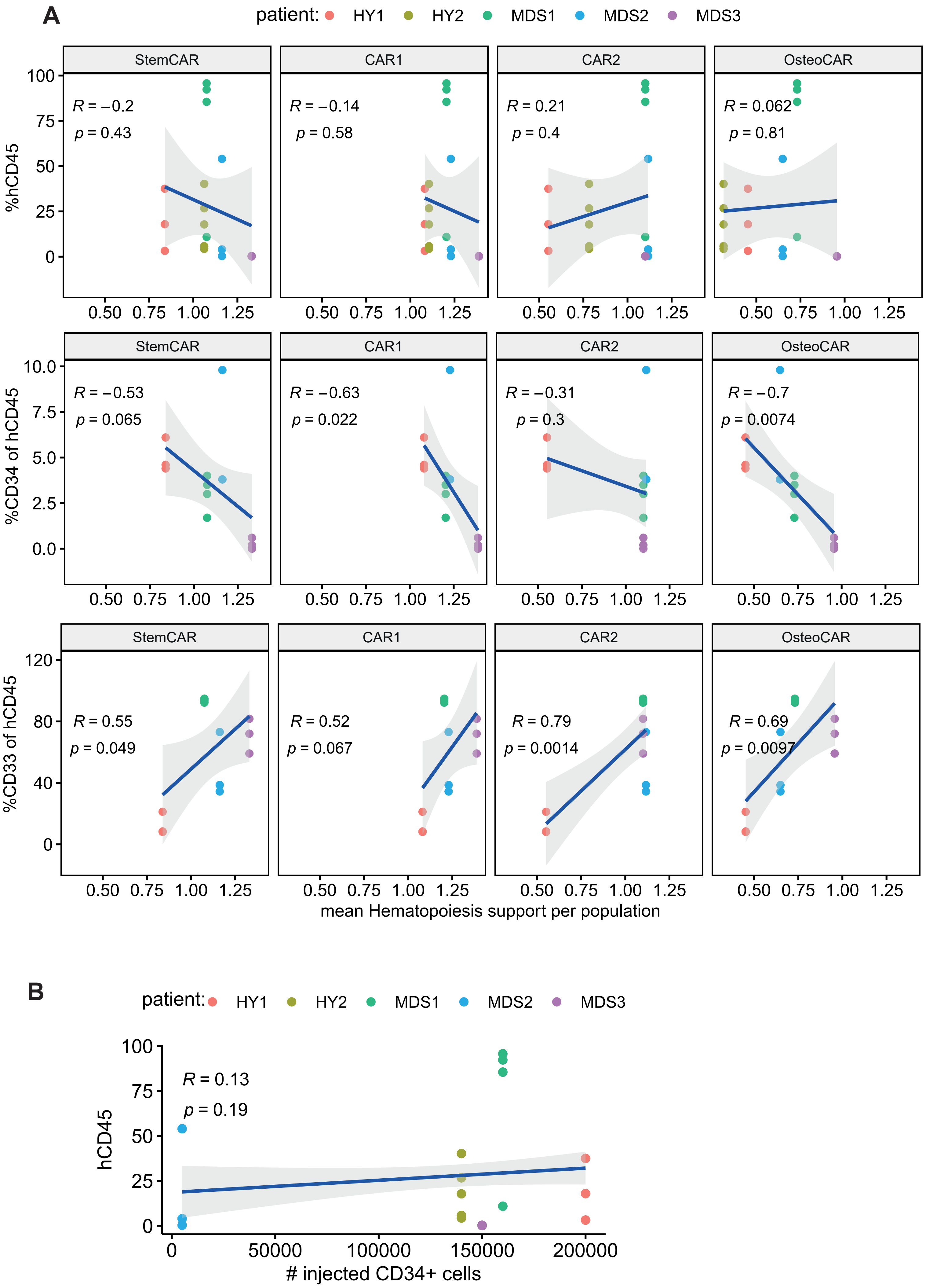

Supplemental Figure 5

A

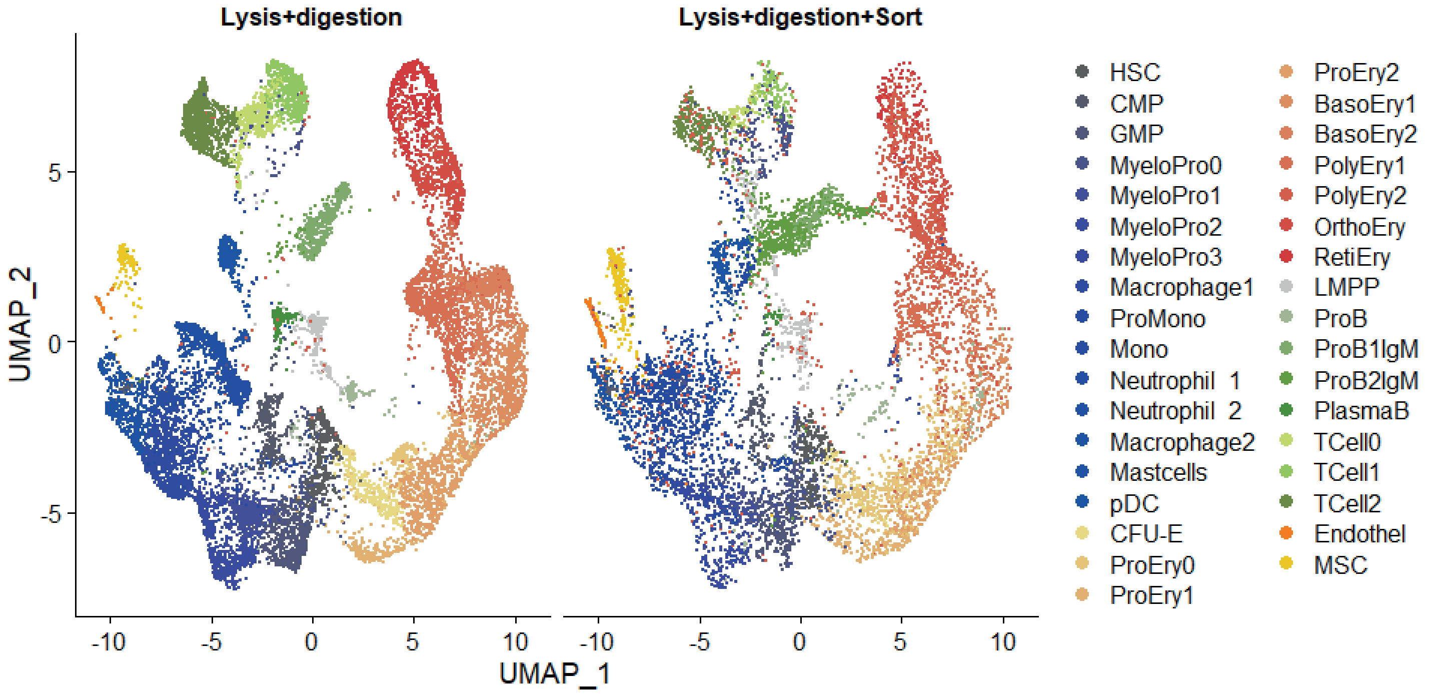

B

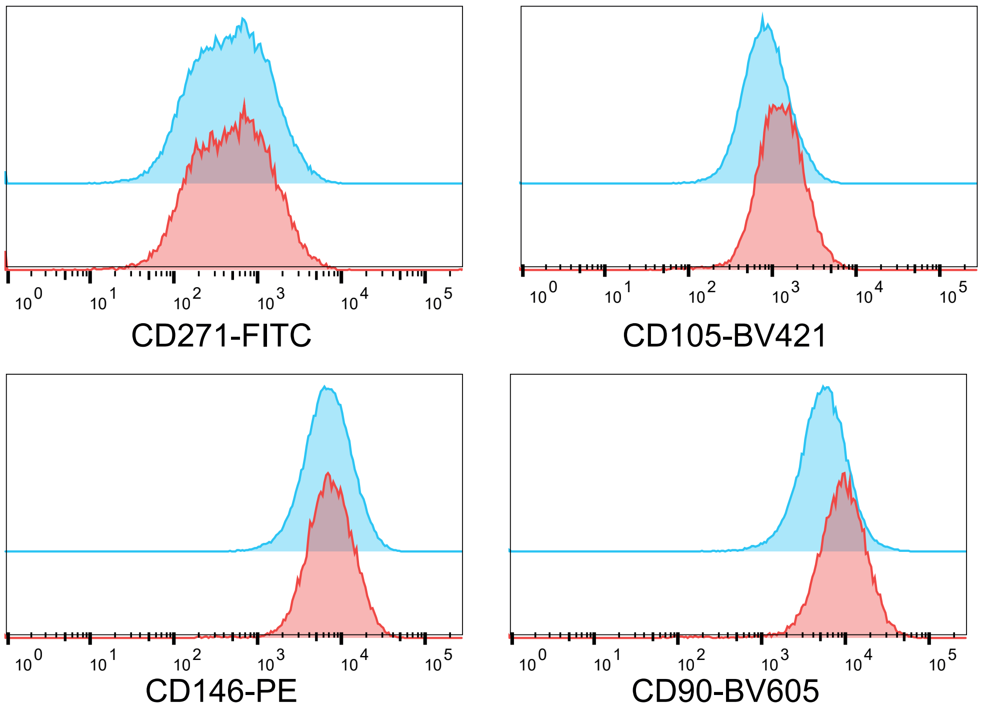

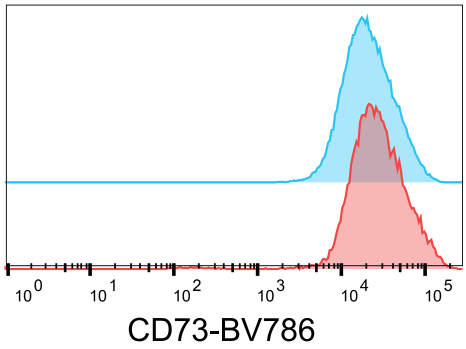

	Sample Name	Subset Name	Count
(blue)	MSC from trephine	lin-	78117
(red)	MSC from aspiration	lin-	24909

Supplemental Figure 6

A

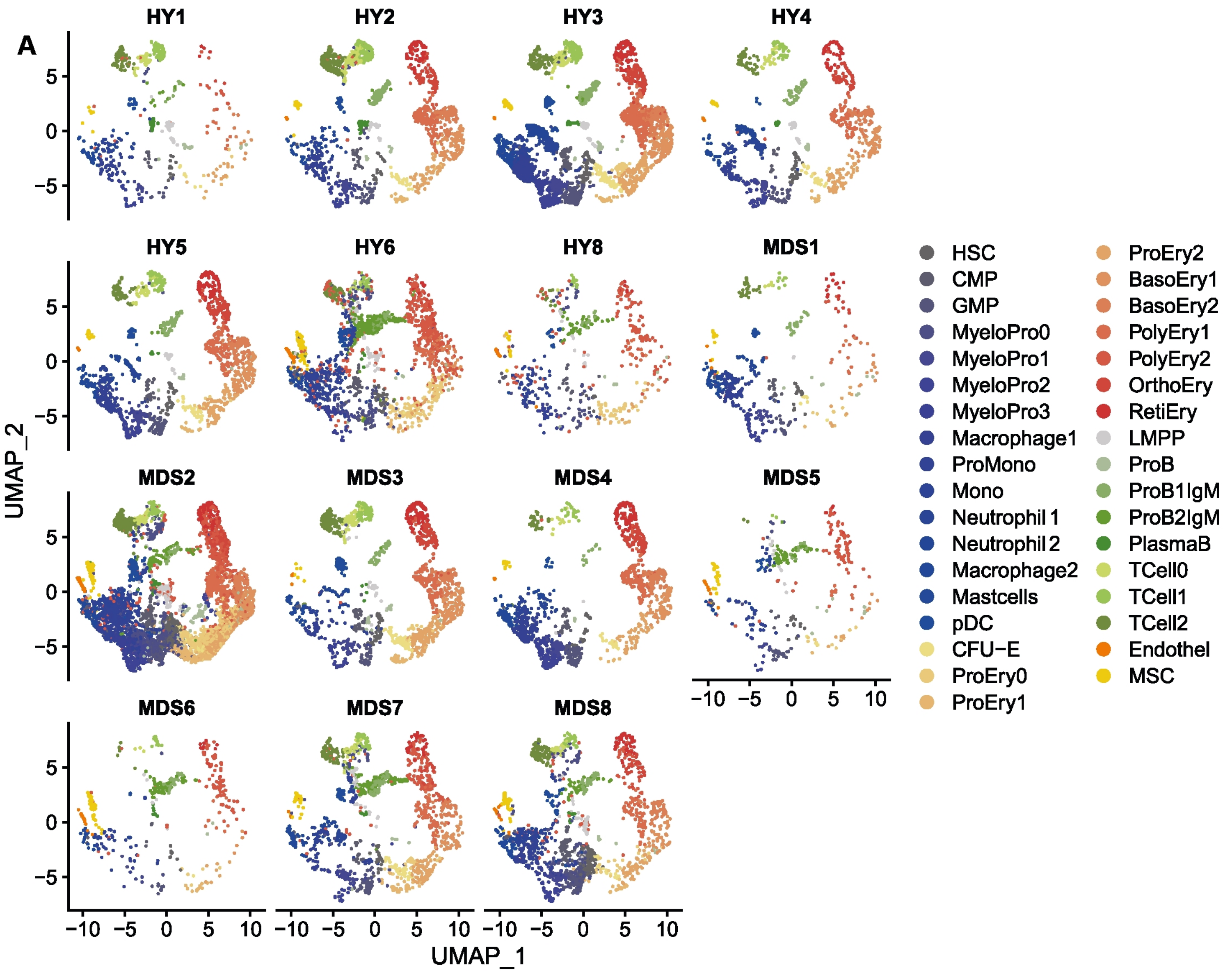

B

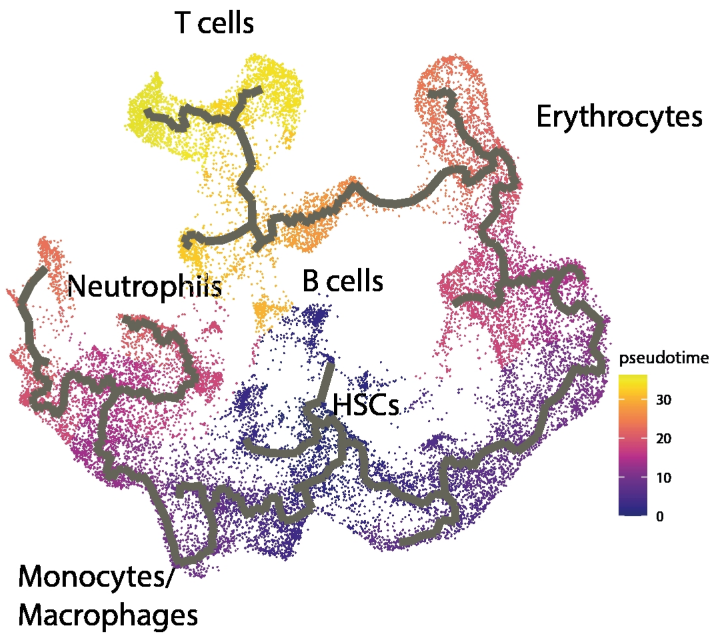

Supplemental Figure 7

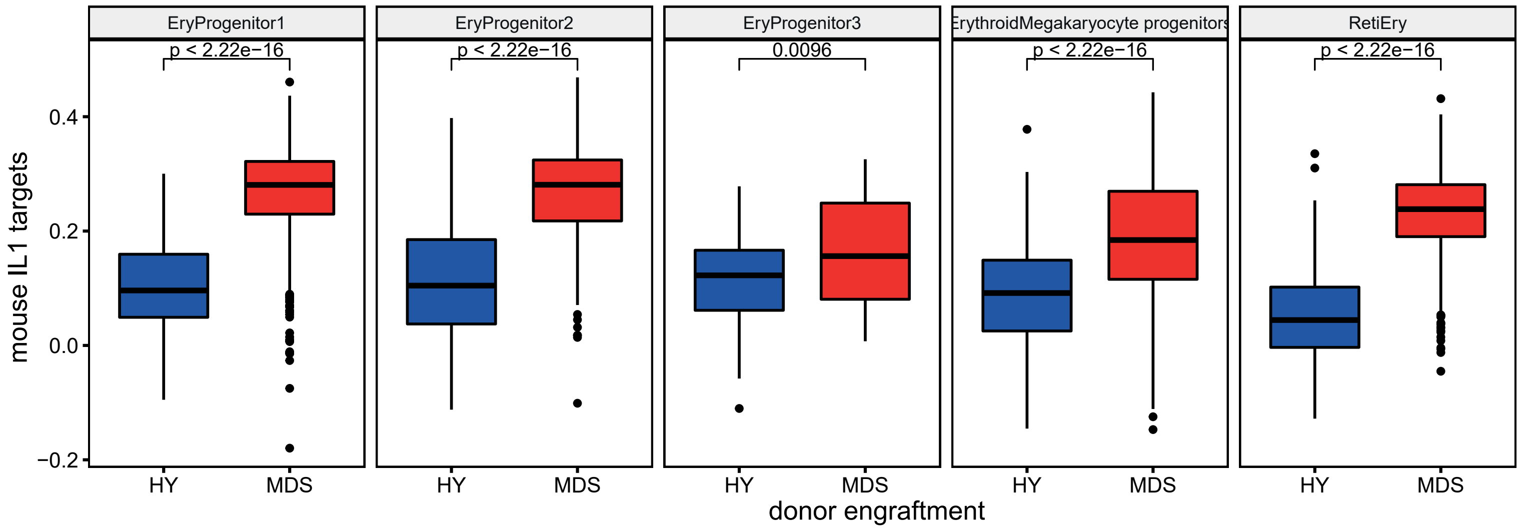

Supplemental Figure 8

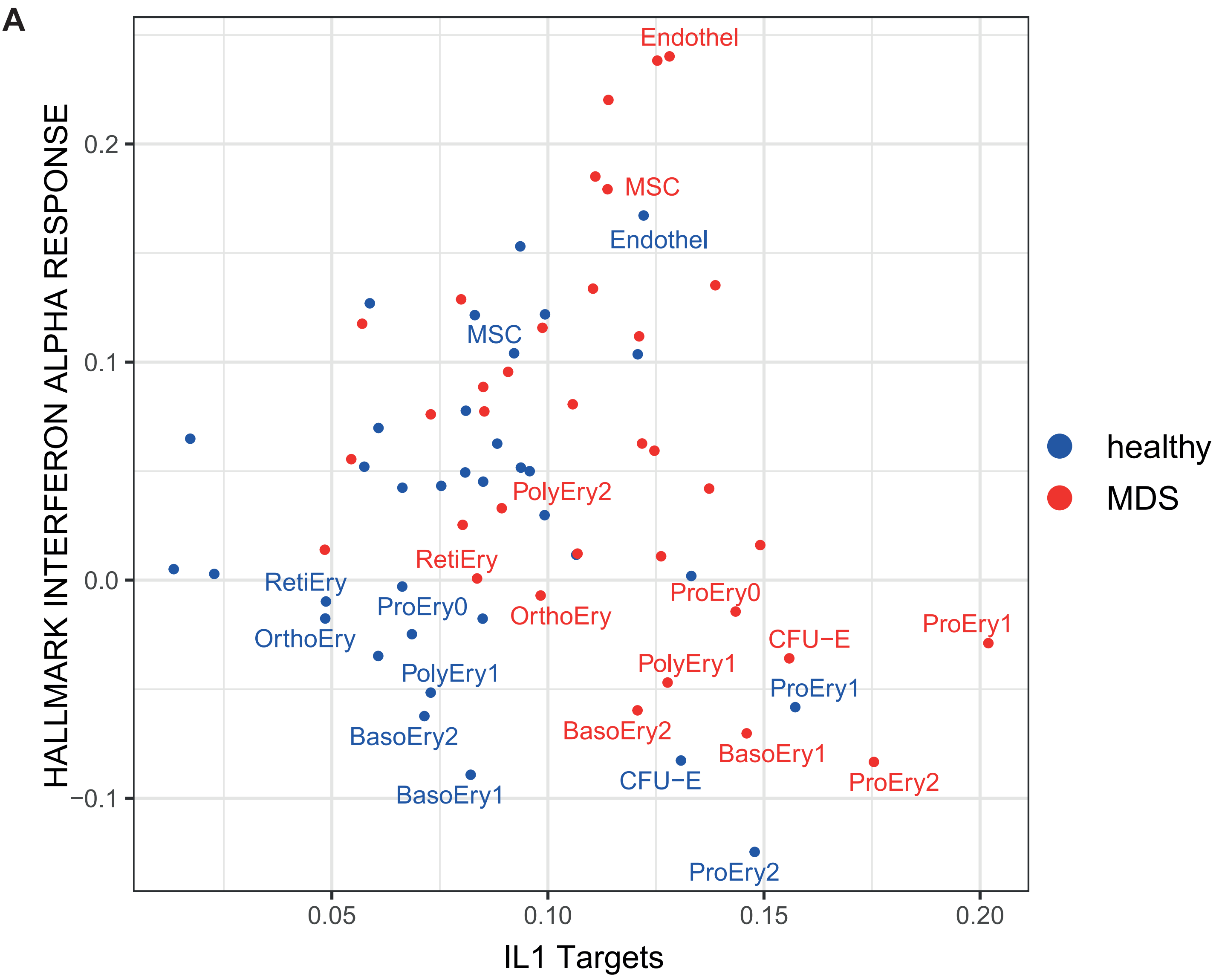

Supplemental Figure 9

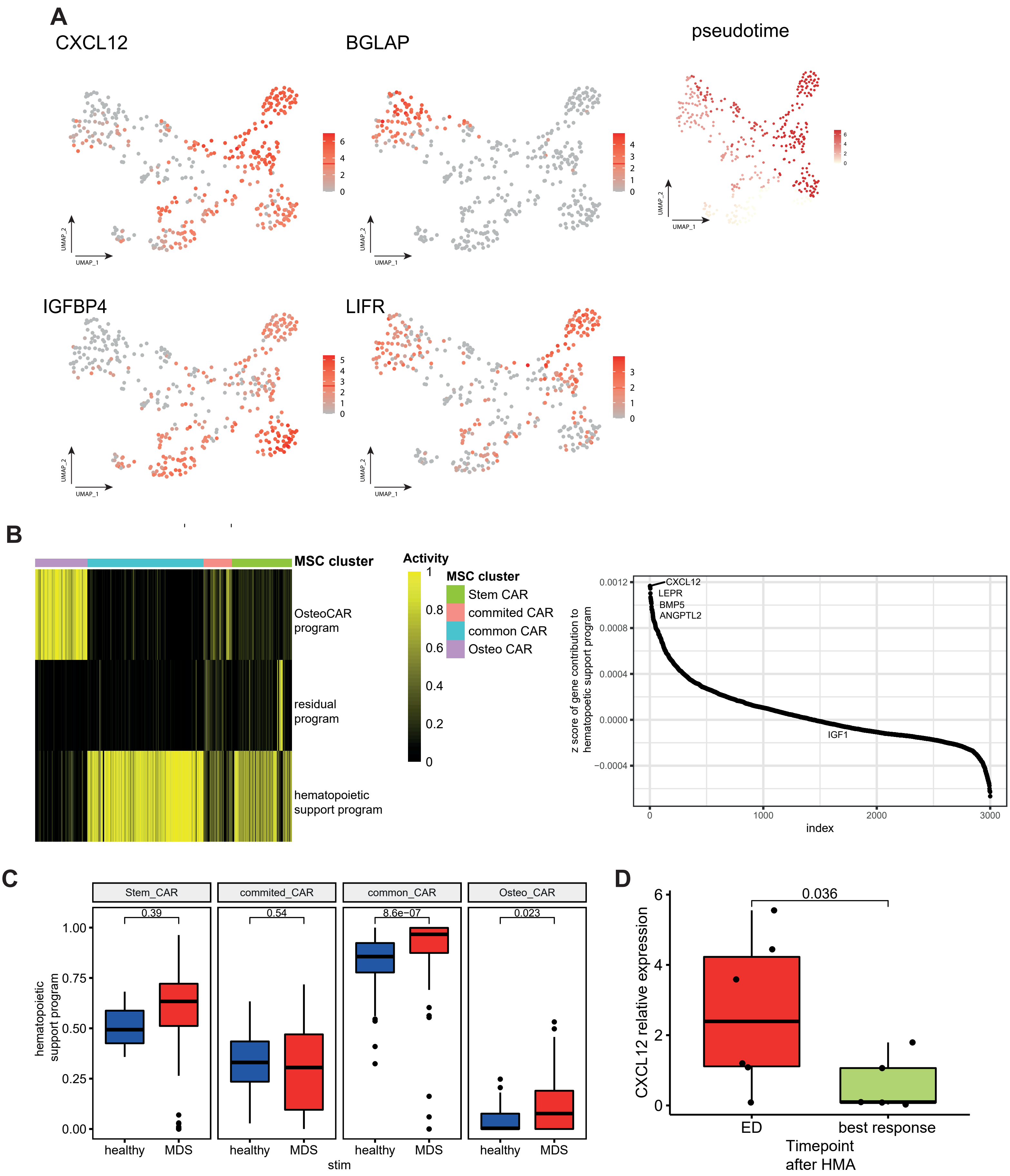

Supplemental Figure 10

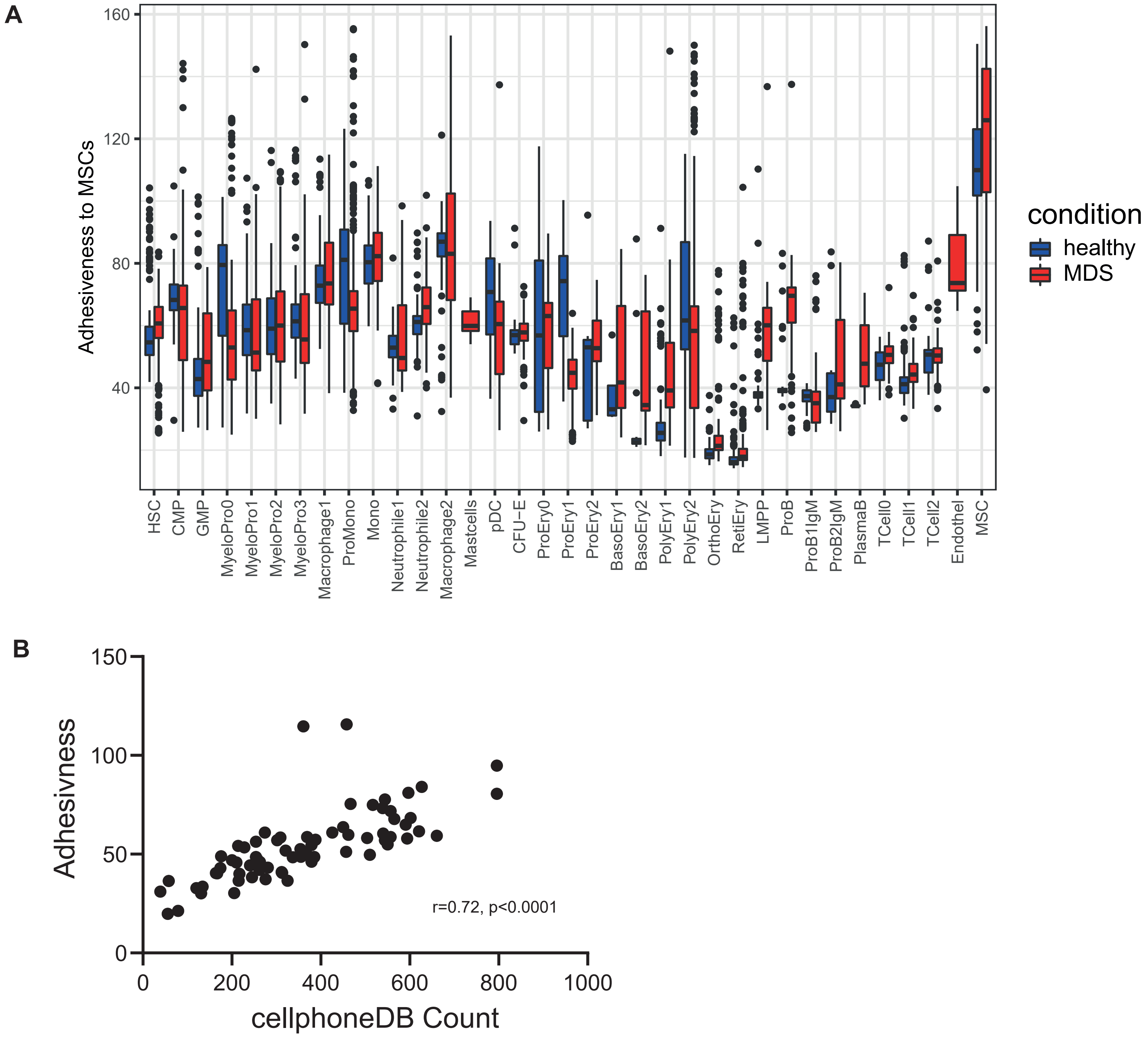

Supplement: Supplemental Figures [file BNEO_NEO-2023-000161-mmc2.pdf]
